# Analytical Model of Nonlinear Stress-Strain Relation for a Strand Made of Two Materials

**DOI:** 10.3390/ma10091003

**Published:** 2017-08-28

**Authors:** Keunhee Cho, Sung Tae Kim, Jeong-Rae Cho, Young-Hwan Park

**Affiliations:** Structural Engineering Research Institute, Korea Institute of Civil Engineering and Building Technology, 283, Goyangdae-Ro, Ilsanseo-Gu, Goyang-Si, Gyeonggi-Do 10223, Korea; esper009@kict.re.kr (S.T.K.); chojr@kict.re.kr (J.-R.C.); yhpark@kict.re.kr (Y.-H.P.)

**Keywords:** strand, smart strand, CFRP, stress-strain relation, nonlinear, optical fiber sensor

## Abstract

Unlike conventional steel strands, the smart strand supports strain-measuring function and adopts different materials for its core wire and helical wires. This study intends to analytically derive the nonlinear stress-strain model of this strand made of two materials. The effect of the bending moment and torsional moment of the helical wires on the overall load within the range of geometric shapes shown by actually used strands is verified to be negligible and is thus ignored in order to simplify the analytical model. Moreover, the slight difference between the actual and analytic behaviors, which only appears in the slope varying part in the case of bilinear behavior, such as that of steel, is also ignored. The proposed constitutive model of the smart strand obtained by introducing the experimental stress-strain relation between the carbon fiber reinforced polymer core wire and the helical steel wires is in good agreement with the experimental data. The previous analytical models are applicable only to strands made of a unique linear material, whereas the model proposed in this study is also applicable to strands in which the core wire and the helical wires are made of two different materials, exhibiting nonlinear behavior.

## 1. Introduction

**Figure 1:**
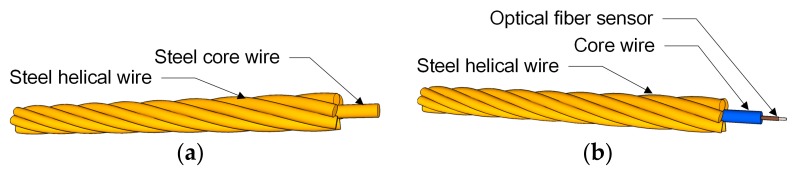
Composition of a seven-wire strand: (**a**) Steel strand; (**b**) Smart strand.

In prestressed concrete (PSC) structures, the jacking force is introduced mainly through the strands. The smart strand was developed recently to be able to measure the prestress force in PSC structures throughout their service life since their erection. The smart strand is achieved by replacing the core wire of the traditional steel strand by a steel tube [1,2], a CFRP (Carbon Fiber-Reinforced Polymer) rod [3], or a GFRP (Glass Fiber-Reinforced Polymer) rod [4] in which an optical fiber sensor is installed (Figure 1). Such an arrangement results in the application of different materials for the core wire and its surrounding helical wires, and necessitates providing a new load-strain model for the smart strand.

Costello [5] and Velinsky [6] conducted studies to analytically derive the load-strain relationship of steel strands. These researchers derived load-strain models for strands made of linear elastic steel and ropes made of bundles of strands, and could obtain results accurately simulating the linear elastic behavior. Other studies also numerically analyzed the behavior of strands and ropes. Shibu et al. [7] investigated the influence of the boundary conditions at the ends of the rope made of bundles of steel strands through numerical analysis. Erdönmez and İmrak [8] considered friction and slip in the analysis of steel strands and ropes. Nawrocki and Labrosse [9] accounted for various conditions (rolling, sliding, pivoting) between the wires of the steel strand. Jiang and Henshall [10] considered the effect of a fixed-end termination on the contact forces in the analysis of inelastic steel strands. All of these studies concerned strands in which the core wire and helical wires are made of steel, and thus they are not applicable in the case of a core wire and helical wires made of different materials. In addition, Jiang and Henshall [10] were the only ones who considered the material nonlinearity in their numerical analysis.

The present study intends to analytically derive the load-strain relationship of strands in which the core wire and helical wires are made of different materials, as is the case for the smart strand. The formulation is conducted for cases where the core wire and helical wires are made of linear materials and nonlinear materials.

## 2. Compatibility of the Strand

This chapter deals with the relation between the geometric parameters of the wire before and after deformation. The adopted process is basically similar to that of Costello [5], but the formulation is conducted to allow different materials for the core wire and helical wires. This implies some difference in the compatibility conditions, considering the different Poisson’s ratios exhibited by the core and helical wires.

The strand presents a straight core wire surrounded by m helical wires. When the radius of the core wire is $R_{1}$ and that of the helical wire is $R_{2}$, the helix radius $r_{2}$ of the helical wire before deformation of the strand is:

(1) $$r_{2}=R_{1}+R_{2}$$

In addition, the initial helix angle $\alpha_{2}$ of the helical wire can be expressed as follows using the helix radius $r_{2}$ and the pitch $p_{2}$:

(2) $$\tan\alpha_{2}=\frac{p_{2}}{2\pi r_{2}}$$

**Figure 2:**
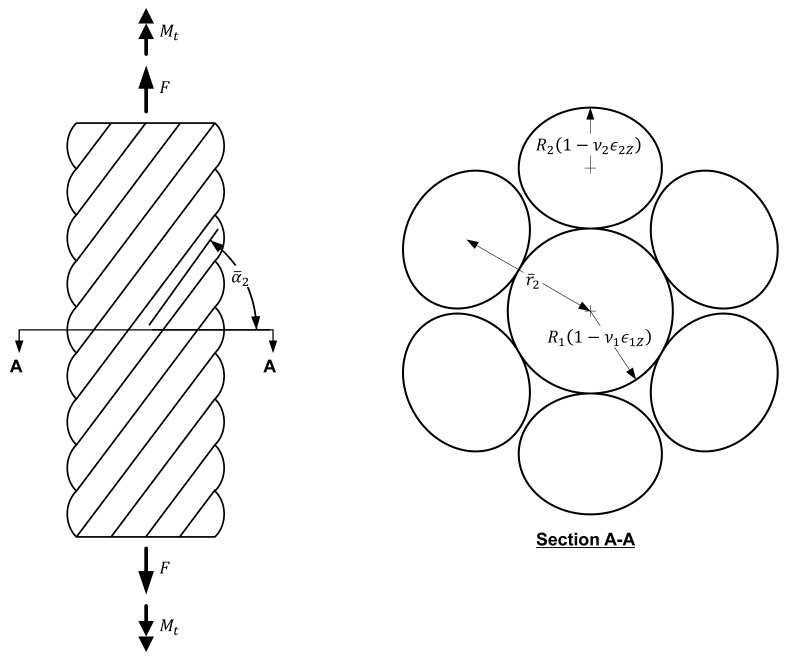
Geometry of a loaded strand.

The strand undergoes deformation according to the application of the load, as shown in Figure 2. Once the strand is loaded, the diameters of the core and helical wires change respectively into $R_{1}\left(1-\nu_{1}\epsilon_{1Z}\right)$ and $R_{2}\left(1-\nu_{2}\epsilon_{2Z}\right)$ due to the Poisson’s effect, and lead to the helix radius (${\bar{r}}_{2}$) of the helical wire becoming $R_{1}\left(1-\nu_{1}\epsilon_{1Z}\right)+R_{2}\left(1-\nu_{2}\epsilon_{2Z}\right)$. Denoting the post-deformation helix angle by ${\bar{\alpha }}_{2}$, the change $\Delta \alpha_{2}$ of the helix angle is:

(3) $$\Delta \alpha_{2}={\bar{\alpha }}_{2}-\alpha_{2}$$

If the strand is straight, then its longitudinal strain $\epsilon_{ps}$ and the longitudinal strain $\epsilon_{1Z}$ of the core wire will be equal. In addition, the corresponding longitudinal strain $\epsilon_{2Z}$ of the helical wire can be expressed as follows:

(4) $$\epsilon_{ps}=\epsilon_{1Z}=\left(1+\epsilon_{2Z}\right)\frac{\sin{\bar{\alpha }}_{2}}{\sin\alpha_{2}}-1$$

The rotational strain $\beta_{2}$ of the helical wire can be expressed as the product of the helix radius of the helical wire, $r_{2}$, and the angle of twist per unit length of strand, $\tau_{s}$, leading to the following equation:

(5) $$\beta_{2}=r_{2}\tau_{s}=\frac{r_{2}}{{\bar{r}}_{2}}\frac{1+\epsilon_{1Z}}{\tan{\bar{\alpha }}_{2}}-\frac{1}{\tan\alpha_{2}}$$

Due to the Poisson’s ratio effect, the helix radius of the helical wire after deformation, ${\bar{r}}_{2}$, becomes:

(6) $${\bar{r}}_{2}=r_{2}-\left(\nu_{1}R_{1}\epsilon_{1Z}+\nu_{2}R_{2}\epsilon_{2Z}\right)$$

where $\nu_{1}$ and $\nu_{2}$ are the Poisson’s ratios of the core wire and helical wire, respectively.

Since $\Delta \alpha_{2}$, $\epsilon_{1Z}$ and $\epsilon_{2Z}$ are very small in Equations (3)–(5), Equations (4) and (5) can be approximated as follows:

(7) $$\epsilon_{1Z}=\epsilon_{2Z}+\frac{\Delta \alpha_{2}}{\tan\alpha_{2}}$$

(8) $$\beta_{2}=r_{2}\tau_{s}=\frac{\epsilon_{2Z}}{\tan\alpha_{2}}-\Delta \alpha_{2}+\frac{\nu_{1}R_{1}\epsilon_{1Z}+\nu_{2}R_{2}\epsilon_{2Z}}{r_{2}\tan\alpha_{2}}$$

Combining Equations (7) and (8), and solving the system of equations gives:

(9) $$\epsilon_{2Z}=\;C_{1}\epsilon_{1Z}+C_{2}$$

(10) $$\Delta \alpha_{2}=\left(1-C_{1}\right)\tan\alpha_{2}\epsilon_{1Z}-C_{2}\tan\alpha_{2}$$

where the coefficients $C_{1}$ and $C_{2}$ are:

(11) $$C_{1}=\frac{r_{2}\tan^{2}\alpha_{2}-\nu_{1}R_{1}}{r_{2}\tan^{2}\alpha_{2}+r_{2}+\nu_{2}R_{2}}$$

(12) $$C_{2}=\frac{r_{2}^{2}\tau_{s}\tan\alpha_{2}}{r_{2}\tan^{2}\alpha_{2}+r_{2}+\nu_{2}R_{2}}$$

Accordingly, the change in curvature with respect to the y-axis, $\Delta \kappa_{2Y}$, as well as the change in twist per unit length, $\Delta \kappa_{2Z}$, can be linearized as follows:

(13) $$\Delta \kappa_{2Y}=-\frac{2\sin\alpha_{2}\cos\alpha_{2}}{r_{2}}\Delta \alpha_{2}+\frac{\nu_{1}R_{1}\epsilon_{1Z}+\nu_{2}R_{2}\epsilon_{2Z}}{r_{2}}\frac{\cos^{2}\alpha_{2}}{r_{2}}$$

(14) $$\Delta \kappa_{2Z}=\frac{1-2\sin^{2}\alpha_{2}}{r_{2}}\Delta \alpha_{2}+\frac{\nu_{1}R_{1}\epsilon_{1Z}+\nu_{2}R_{2}\epsilon_{2Z}}{r_{2}}\frac{\sin\alpha_{2}\cos\alpha_{2}}{r_{2}}$$

## 3. Load-Strain Relation

**Figure 3:**
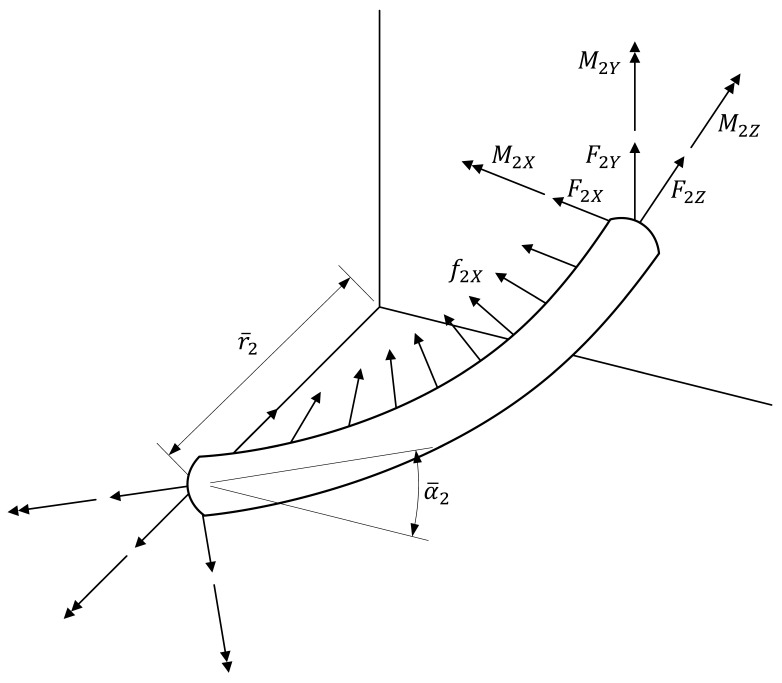
Loads acting on a helical wire.

This chapter derives the load-strain relation for both linear and nonlinear materials. Figure 3 presents the components of the load applied to the helical wire.

### 3.1. Linear Materials

This section derives the load-strain relation when the core wire and helical wire are made of different linear materials.

The moment with respect to the y-axis of the helical wire, $M_{2Y}$, and the torsional moment with respect to the longitudinal z-axis of the helical wire, $M_{2Z}$, can be expressed as follows:

(15) $$M_{2Y}=E_{2}I_{2Y}\Delta \kappa_{2Y}$$

(16) $$M_{2Z}=G_{2}I_{2P}\Delta \kappa_{2Z}$$

where $E_{2}$ and $G_{2}$($=\frac{E_{2}}{2\left(1\;+\;\nu_{2}\right)}$) are the elastic modulus and the shear elastic modulus of the helical wire, respectively; and $I_{2Y}$($=\frac{\pi R_{2}^{4}}{4}$) and $I_{2P}$($=\frac{\pi R_{2}^{4}}{2}$) are the moment of inertia and the polar moment of inertia with respect to the y-axis of the helical wire, respectively. These moments enable one to obtain the component $F_{2Y}$ of the section force in the y-axis of the helical wire.

(17) $$F_{2Y}=M_{2Z}\frac{\cos^{2}\alpha_{2}}{r_{2}}-M_{2Y}\frac{\sin\alpha_{2}\cos\alpha_{2}}{r_{2}}$$

The component $F_{2Z}$ of the section force in the longitudinal axis of the helical wire is:

(18) $$F_{2Z}=E_{2}A_{2}\epsilon_{2Z}$$

where $A_{2}$ is the cross-sectional area of one helical wire. Summing up the components of the section force of the helical wire in the longitudinal direction of the strand for all the helical wires gives the resisting force $F_{2}$ acting on all the helical wires in the longitudinal direction of the strand.

(19) $$F_{2}=m_{2}\left(F_{2Z}\sin\alpha_{2}+F_{2Y}\cos\alpha_{2}\right)$$

The component $F_{1Z}$ of the section force in the longitudinal direction of the core wire is equal to the resistance force $F_{1}$ of the core wire along the length of the strand, and can be obtained as follows:

(20) $$F_{1}=F_{1Z}=E_{1}A_{1}\epsilon_{1Z}$$

The force F acting on the strand is obtained as the sum of the resistance forces acting in the core and helical wires.

(21) $$F=F_{1}+F_{2}$$

In order to evaluate the contribution of each component of the force acting on the strand, Equation (21) is rearranged as follows by separating the components:

(22) $$F=F_{1Z}+m_{2}F_{2Z}\sin\alpha_{2}-m_{2}M_{2Y}\frac{\sin\alpha_{2}\cos^{2}\alpha_{2}}{r_{2}}+m_{2}M_{2Z}\frac{\cos^{3}\alpha_{2}}{r_{2}}$$

where the contributing percentage of each term to the force acting on the strand is listed in Table 1 by using the actual shape range of the seven-wire strand with a diameter of 15.2 mm ($2.56\;\mathrm{mm}\leq R_{1}\leq 2.60\;\mathrm{mm}$, $2.50\;\mathrm{mm}\leq R_{2}\leq 2.52\;\mathrm{mm}$, $182.4\;\mathrm{mm}\leq p_{2}\leq 273.6\;\mathrm{mm}$). In Equation (22), the first term $F_{1Z}$ and the second term $m_{2}F_{2Z}\sin\alpha_{2}$ contribute 15–16% and 84–85% to the strand force, respectively. Meanwhile, the third and fourth terms have nearly 0% contribution to the strand force.

Let us now see the effect of the rotation of the strand on each term of Equation (22). Table 2 arranges the contribution of each term of Equation (22) on the strand force in occurrence of rotations by 90 degrees and −90 degrees per meter of the steel strand. Similar to Table 1, it appears that the contributions of the third and fourth terms of Equation (22) are practically null.

From an engineering standpoint, the third and fourth terms can be ignored, and the strand force F can be replaced as follows by the simplified force $F_{S}$:

(23) $$F_{S}=E_{1}A_{1}\epsilon_{1Z}+m_{2}E_{2}A_{2}\epsilon_{2Z}\sin\alpha_{2}$$

### 3.2. Nonlinear Materials

The possibility to simplify the force acting on the strand by summing up the force components in the longitudinal direction of the core and helical wires was verified in the precedent section. The present section intends to obtain this force by means of a simplified equation in the case where the core and helical wires are made of different, nonlinear materials.

First, the force of the core wire can be obtained by integrating the stress $\sigma_{1Z}\left(\epsilon_{1Z}\right)$ developed in the core wire over its cross-sectional area.

(24) $$F_{1Z}=\int_{A_{1}}^{}\sigma_{1Z}\left(\epsilon_{1Z}\right)dA$$

Since the strain is constant over the cross-sectional area of the core wire, Equation (24) can be rewritten as follows:

(25) $$F_{1Z}=A_{1}\sigma_{1Z}\left(\epsilon_{1Z}\right)$$

Besides, the strain in the helical wire can be expressed as $\epsilon_{2Z}-x\Delta \kappa_{2Y}$, where $\epsilon_{2Z}$ is the strain along the length of the helical wire and $x\Delta \kappa_{2Y}$ corresponds to the change in the curvature relative to the y-axis of the helical wire, in which x is the distance from the center of the cross-section of the helical wire in the x-axis direction. Similar to the core wire, the force of the helical wire can also be obtained by integrating the stress $\sigma_{2Z}\left(\epsilon_{2Z}-x\Delta \kappa_{2Y}\right)$ over its cross-sectional area.

(26) $$F_{2Z}=\int_{A_{2}}^{}\sigma_{2Z}\left(\epsilon_{2Z}-x\Delta \kappa_{2Y}\right)dA$$

**Figure 4:**
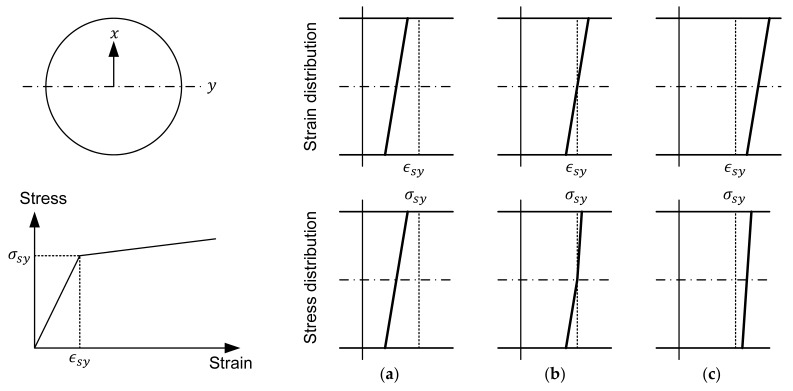
Stress distribution according to strain distribution in the longitudinal direction of helical wire: (**a**) before yielding; (**b**) during yielding; (**c**) after yielding of helical wire.

Because of the symmetry of the helical wire with respect to the y-axis, $x\Delta \kappa_{2Y}$ will not have any effect on $F_{2Z}$ in the case of a linear stress-strain relation in the section. Figure 4 depicts the distribution of the longitudinal strain together with the stress distribution inside the cross-section of the helical wire. Considering a bilinear behavior for the material of the helical wire, the stress will also exhibit linear distribution, as expressed below, when the strain range inside the cross-section is smaller than the yield strain $\epsilon_{sy}$ as in (a), or is larger than $\epsilon_{sy}$ as in (c).

(27) $$\int_{A_{2}}^{}\sigma_{2Z}\left(\epsilon_{2Z}-x\Delta \kappa_{2Y}\right)dA≅\int_{A_{2}}^{}\sigma_{2Z}\left(\epsilon_{2Z}\right)dA$$

Note that Equation (27) does not hold in the part where the range of the strain in the cross-section includes the yield strain, as in (b). However, $\left|R_{2}\Delta \kappa_{2Y}\right|$ runs around 1.5% to 3.4% of $\left|\epsilon_{2Z}\right|$ for the actual shape range of the seven-wire strand with a diameter of 15.2 mm ($2.56\;\mathrm{mm}\leq R_{1}\leq 2.60\;\mathrm{mm}$, $2.50\;\mathrm{mm}\leq R_{2}\leq 2.52\;\mathrm{mm}$, $182.4\;\mathrm{mm}\leq p_{2}\leq 273.6\;\mathrm{mm}$). This represents the largest difference that could occur due to $\left|x\Delta \kappa_{2Y}\right|$. This difference becomes smaller at the center of the section where the contribution to $F_{2Z}$ is higher, as the cross-sectional area is larger. Consequently, the effect of $x\Delta \kappa_{2Y}$ is ignored here, since it induces difference only in the limited portion where the stress distribution is not linear. Accordingly, $F_{2Z}$ of Equation (26) can be replaced by $F_{2Z,S}$ without integration, shown as follows:

(28) $$F_{2Z,S}=\int_{A_{2}}^{}\sigma_{2Z}\left(\epsilon_{2Z}\right)dA=A_{2}\sigma_{2Z}\left(\epsilon_{2Z}\right)$$

Finally, the load-strain relation of the strand made of nonlinear materials resulting from this process can be obtained as follows:

(29) $$F_{S}=A_{1}\sigma_{1Z}\left(\epsilon_{1Z}\right)+m_{2}A_{2}\sigma_{2Z}\left(\epsilon_{2Z}\right)\sin\alpha_{2}$$

Substituting the linear material models of the core and helical wires in Equation (29) results in the load-strain model for linear materials in Equation (23).

## 4. Verification

Comparison is done with actual experimental data to validate the established load-strain relation.

**Figure 5:**
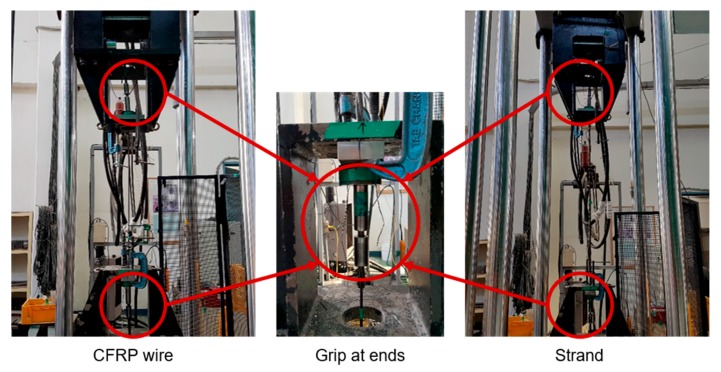
View of tensile test conducted on CFRP (Carbon Fiber-Reinforced Polymer) wire, steel strand, and smart strand.

The stress-strain relation of the materials used for the core and helical wires constituting the strand is necessary to apply the proposed model in Equation (29). The smart strand adopted for the verification in this study is composed a CFRP core wire and steel helical wires. Material models are thus necessary for the CFRP wire and steel wire. To that end, tensile tests were conducted as shown in Figure 5 for the CFRP wire and steel strand. In addition, tensile test was also performed on the smart strand itself to validate the proposed method. As described in Figure 5, both ends of the steel strand were fixed by pressure grips to prevent sliding between the core wire and the helical wires. Two grips were installed at the ends of the CFRP wire wrapped by six helical wires. T700 carbon fiber of Toray Industries is used for the CFRP wire. Note that the volume fraction of the CFRP wire is 80%.

The following stress-strain relation was obtained from the tensile test of the CFRP wire. Here, the strain is derived from the FBG (Fiber Bragg Grating) wavelength of the optical fiber embedded in the CFRP wire:

(30) $$\sigma_{CFRP}\left(\epsilon \right)=E_{CFRP}\epsilon$$

where the elastic modulus ($E_{CFRP}$) of the CFRP core wire is 173 GPa.

**Figure 6:**
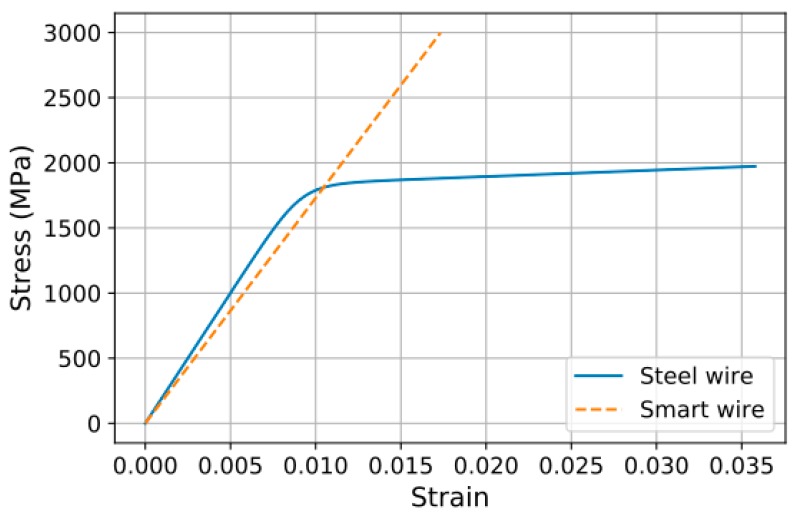
Material models for steel wire and CFRP wire.

The results of the direct tensile test of the steel strand were used to indirectly derive those of the steel wires. In other words, the stress-strain relation of the steel wire was obtained by solving the following optimization problem to minimize the difference between the experimental curve of the steel strand and Equation (29):

(31) $$\min\overset{n}{\underset{i}{\sum }}{\left(F_{s,i}-F_{e,i}\right)}^{2}\;,\;i=1,\;\ldots ,\;n$$

where n is the number of data; $F_{e,i}$ is the i^th^ load value in the test; and $F_{s,i}$ is the value of the load obtained by substituting the i^th^ strain of the test in Equation (29). The bilinear stress-strain model of the steel material was applied by Menegotto [11], Hoehler and Stanton [12], and other researchers for repeated loading, as well as by Mattock [13] for monotonically increasing loading. Since the material model for monotonically increasing load is sufficient, this study adopts the comparatively simple model of Mattock, expressed in Equation (32).

(32) $$\sigma_{s}\left(\epsilon_{s}\right)=E_{s}\epsilon_{s}\left(A+\frac{\left(1-A\right)}{{\left[1+{\left(B\epsilon_{s}\right)}^{C}\right]}^{1/C}}\right)$$

where $\sigma_{s}$ is the stress; $\epsilon_{s}$ is the strain; and $E_{s}$, A, B, C are material model constants, for which values resulting from the optimization are 200 GPa, 0.025, 109, and 10.8, respectively. Figure 6 plots the smart wire (CFRP wire) and steel wire.

The substitution of these material models for the CFRP wire and steel wire into Equation (29) provides the load-strain relations of the steel strand and smart strand. In the steel strand, the diameter of the core wire ($R_{1}$) is 2.6 mm and that of the helical wire ($R_{2}$) is 2.51 mm. The Poisson’s ratio of the core and helical wires ($\nu_{1}$, $\nu_{2}$) is 0.3, and the lay length of the steel strand ($p_{2}$) is 225 mm. In the smart strand, the core wire has a diameter ($R_{1}$) of 2.65 mm with a Poisson’s ratio ($\nu_{1}$) of 0.3, and the helical wire is identical to that of the steel strand.

**Figure 7:**
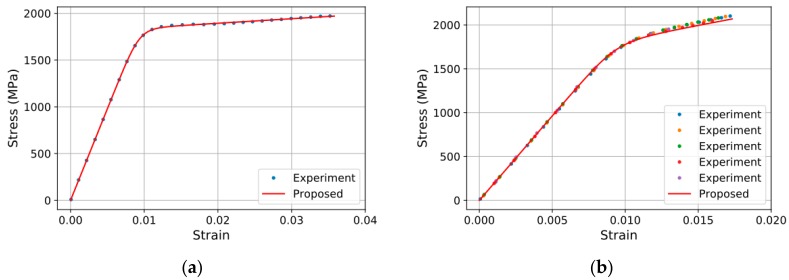
Comparison of experimental and analytical results: (**a**) steel strand; (**b**) smart strand.

The comparison of the analytical and experimental results in Figure 7 reveals their good agreement. The concordance observed for the steel strand is obvious since the material model of the steel wire used in the analytical formula for the steel strand was derived based upon the experimental results of the steel strand. Moreover, good agreement is also observed for the smart strand even if the analytical results for the smart strand were obtained by substituting the material models of the CFRP core wire and steel wire derived individually. These results demonstrate the feasibility and validity of the method deriving the load-strain model of strands made of two materials proposed in this study.

## 5. Conclusions

The load-strain model of the strands was analytically derived considering the case where the core and helical wires are made of different materials, such as in the smart strand. This model can be applied not only for linear materials but also for nonlinear materials. Moreover, all the parameters determining the shape of the strands, such as the diameter of the core and helical wires and the pitch, can be considered.

The effect of the bending moment and torsional moment of the helical wires on the overall load within the range of geometric shapes shown by actually used strands was verified to be negligible, and was thus ignored in order to simplify the analytical model. Moreover, the slight difference between the actual and analytic behaviors, appearing only in the slope varying part in the case of bilinear behavior, such as that of steel, was also ignored. The proposed constitutive model of the smart strand obtained by introducing the experimental stress-strain relation between the CFRP core wire and the helical steel wires was in good agreement with the experimental data.

The previous analytical models are applicable only to strands made of a unique linear material, whereas the model proposed in this study is also applicable to strands in which the core wire and the helical wires are made of two different materials, exhibiting nonlinear behavior.

## Figures and Tables

**Table 1:** Contribution of each term of Equation (22) in the shape change of the steel strand.

Shape (mm)			Contributing Percentage to Strand Force (%)			
$R_{1}$	$R_{2}$	$p_{2}$	First Term	Second Term	Third Term	Fourth Term
2.56	2.52	182.4	15.4	84.6	0.0	0.0
2.56	2.52	273.6	15.0	85.0	0.0	0.0
2.60	2.50	182.4	16.0	84.0	0.0	0.0
2.60	2.50	273.6	15.6	84.4	0.0	0.0

**Table 2:** Contribution of each term of Equation (22) in the case of rotation of the steel strand.

Rotation (Degree/m)	Contributing Percentage to Strand Force (%)			
	First Term	Second Term	Third Term	Fourth Term
90	14.4	85.7	0.0	0.0
−90	17.5	82.5	0.0	0.0
